# Dietary isoflavone and the risk of colorectal adenoma: a case–control study in Japan

**DOI:** 10.1038/sj.bjc.6605088

**Published:** 2009-05-05

**Authors:** M Akhter, M Iwasaki, T Yamaji, S Sasazuki, S Tsugane

**Affiliations:** 1 Epidemiology and Prevention Division, Research Center for Cancer Prevention and Screening, National Cancer Center, Tokyo, Japan

**Keywords:** isoflavone, colorectal adenoma, case–control study, Japan

## Abstract

We conducted a case–control study in a Japanese population to investigate the association between dietary isoflavone intake and the risk of colorectal adenoma. Participants who underwent magnifying colonoscopy with dye spreading as part of a cancer screening programme responded to a self-administered questionnaire, which included lifestyle information and intake of 145 food items, before the colonoscopy. A total of 721 case and 697 control subjects were enrolled. Odds ratios (ORs) and 95% confidence intervals (CIs) were estimated using unconditional logistic regression models. We found a significant inverse association between dietary isoflavone intake and the risk of colorectal adenoma in men and women combined. However, the inverse association was not linear; rather, all quartiles above the first showed a similar decrease in risk, with multivariable-adjusted ORs and 95% CIs compared with the lowest quartile of 0.77 (0.57–1.04), 0.76 (0.56–1.02) and 0.70 (0.51–0.96) in the second, third and highest quartiles, respectively (*P* for trend=0.03). Of interest, the observed association was more prominent in women than in men. The observed ceiling effect associated with higher isoflavone intake suggests that a lower intake of dietary isoflavone might be associated with an increased risk of colorectal adenoma in Japanese populations.

The incidence of colorectal cancer, a common malignancy in developed countries, is higher in men than in women (Parkin *et al*, 2002), a difference which has been related to sex hormones, in particular oestrogen. Although the function of oestrogen in the aetiology of colorectal cancer has not been established, an association is supported by several studies (MacLennan *et al*, 1995; Hébert-Croteau, 1998; Grodstein *et al*, 1999; Chlebowski *et al*, 2004; Linsalata *et al*, 2005).

Isoflavones such as genistein and daidzein are classified as phyto-oestrogens. Dietary phyto-oestrogens are plant-derived, non-steroidal compounds with weak oestrogen-like activity, occurring predominantly in soya beans and soya products. A better understanding of the association between dietary soya foods or isoflavone intake and colorectal cancer risk would be useful.

To date, seven studies have investigated the association between soya product consumption and the risk of colorectal cancer or adenoma (Hoshiyama *et al*, 1993; Witte *et al*, 1996; Marchand *et al*, 1997; Ho *et al*, 2006; Wakai *et al*, 2006; Oba *et al*, 2007; Akhter *et al*, 2008), of which five reported an inverse association. In contrast, only three studies have assessed the association of isoflavone intake with colorectal cancer risk (Cotterchio *et al*, 2006; Oba *et al*, 2007; Akhter *et al*, 2008), and we are unaware of any study of colorectal adenoma.

We have investigated the possible association between dietary isoflavone intake and the risk of colorectal adenoma, in a case–control study in a Japanese population, who were likely to consume relatively large amounts of isoflavones with substantial variation.

## Materials and methods

The Colorectal Adenoma Study in Tokyo (CAST) consisted of examinees who underwent magnifying colonoscopy with dye spreading as a part of a cancer screening programme provided by the Research Center for Cancer Prevention and Screening, National Cancer Center, between February 2004 and February 2005. Details of this programme have been provided elsewhere (Otani *et al*, 2006; Yamaji *et al*, 2009). Eligible examinees were men aged 50–79 years or women aged 40–79 years who underwent total colonoscopy from the anus to the caecum. Those with a history of any of the following diseases and conditions were considered ineligible: colorectal adenoma, malignant neoplasm, ulcerative colitis, Crohn's disease, familial adenomatous polyposis, carcinoid tumour or colectomy. All subjects gave written informed consent, and the study protocol was approved by the institutional review board of the National Cancer Center, Tokyo, Japan.

Among a consecutive series of 3212 examinees, 2234 met the eligibility criteria above. According to pit-pattern classification (Sano *et al*, 2005), 526 men and 256 women were determined to have at least one adenoma. Of the remaining 1452 examinees, 482 men and 721 women were also free from other benign lesions (e.g. hyperplastic polyps, inflammatory polyps and diverticulum) and were identified as potential controls. The potential control of 256 women was frequency matched to the female case in five age categories (40–49, 50–54, 55–59, 60–64 and ⩾65 years old) and screening periods (first and second half). Because there were fewer potential male controls than male cases, all men of the potential controls were included in the study. Finally, the CAST consisted of 782 cases and 738 controls.

We conducted a self-administered questionnaire survey among participants before their screening examination. The questionnaire assessed demographic characteristics, personal and family medical history, use of drugs and supplements, anthropometric measurements, physical activity, history of smoking and alcohol drinking, reproductive factors (women only) and other lifestyle-related factors.

The self-administered questionnaire included a food frequency questionnaire (FFQ) that asked about 145 food and beverage items in terms of standard portion sizes and 9 frequency categories. The standard portion sizes of respective food items were specified in the FFQ and the amounts were determined into three categories of less than half, the same as, and more than 1.5 times the reference portion size. The frequency response choices for food items were less than once per month, 1–3 times per month, 1–2 times per week, 3–4 times per week, 5–6 times per week, once per day, 2–3 times per day, 4–6 times per day and ⩾7 times per day. Soya food items included *miso* (fermented soya bean paste) soup, *tofu* (soya bean curd) in miso soup, *tofu* (boiled or cold) in other dishes, *atsuage* (fried tofu), *koyadofu* (freeze-dried tofu), *aburaage* (deep-fried tofu), *natto* (fermented soya bean) and *tonyu* (soya milk made from soya bean as major ingredient). The frequency of miso soup consumption was divided into six categories of almost never, 1–3 days per month, 1–2 days per week, 3–4 days per week, 5–6 days per week and daily. Portion size was specified with one bowl of miso soup equalling 150 ml into the nine categories of <1 bowl per day, 1 bowl per day, 2 bowls per day, 3 bowls per day, 4 bowls per day, 5 bowls per day, 6 bowls per day, 7–9 bowls per day and ⩾10 bowls per day. Daily intake of genistein and daidzein was calculated using values in a specially developed food composition table for isoflavone in Japanese foods (Kimira *et al*, 1998; Arai *et al*, 2000). This FFQ was modified from an FFQ used in a previous population-based prospective study that had additional food items, the validity and reproducibility of which were evaluated previously (Tsubono *et al*, 1996; Tsugane *et al*, 2003). Validity was assessed among subsamples of that prospective study using 14- or 28-day dietary records. Spearman's correlation coefficients between the energy-adjusted intake of isoflavone from the questionnaire and dietary records were 0.60 (daidzein) and 0.59 (genistein). Moreover, Spearman's correlation coefficients for daidzein and genistein between energy-adjusted intake from the FFQ and those from serum concentrations were 0.26 and 0.22, respectively, and with those from creatinine-adjusted urinary excretion were 0.40 and 0.30, respectively (Yamamoto *et al*, 2001).

### Statistical analysis

After the exclusion of 102 subjects who reported extreme total energy intake (upper and lower 2.5%) or submitted incomplete answers regarding dietary isoflavone intake and other confounding variables, 721 cases (492 men and 229 women) and 697 controls (460 men and 237 women) were entered into the analysis. Because dietary genistein and daidzein intakes were highly correlated (Spearman *r*=0.99), the sum of genistein and daidzein is provided here as representative for total isoflavones. Isoflavone intake was then adjusted for total energy intake using the residual method in the statistical model after log transformation (Kipnis *et al*, 1993; Brown *et al*, 1994; Willett *et al*, 1997). Participants were divided into quartiles of isoflavone intake based on the distribution of all control men and women. An unconditional logistic regression model was used to calculate odds ratios (ORs) and 95% confidence intervals (95% CIs) of colorectal adenoma for isoflavone intake using the lowest quartile of intake as the referent category. In addition, ORs and 95% CIs were calculated in subgroups stratified by adenoma site (proximal colon, distal colon and rectum), size (<5 and ⩾5 mm) and number (1 and >1). The first statistical model was adjusted for age, sex and screening period, whereas the second model was further adjusted for family history of colorectal cancer (yes or no), cigarette smoking (never, ever <20, ever 21–40, ever >40 pack-years), alcohol drinking (never, ever <150, ever 150–299, ever ⩾300 g per week ethanol), body mass index (<21, 21–22, 23–24, ⩾25 kg m^−2^), physical activity (quartiles based on controls), supplement use (yes or no), non-steroidal anti-inflammatory drug use (yes or no). Age at menarche (<12, 12–13, ⩾14 years), menopausal status (pre or post) and current use of female hormones (user or non-user) were also adjusted in women. Statistical significance of the interaction terms was evaluated by the likelihood ratio test. Tests for linear trend were estimated using the median value of isoflavone intake and treating this variable as continuous. All statistical tests were two tailed, and *P*-values <0.05 were considered statistically significant. All analyses were performed using Stata Statistics/Data Analysis Special Edition version 9.2 (2007; StataCorp LP, College Station, TX, USA).

## Results

The demographic characteristics and lifestyle factors according to case–control and sex had mentioned previously (Otani *et al*, 2006; Yamaji *et al*, 2009). Table 1 shows these factors of controls according to quartile categories of isoflavone intake. Median (interquartile range) isoflavone intake (mg per day) among the control group was 39.73 (24.77–62.41). The proportion of women and supplement users, and mean intake of total isoflavone were higher in the highest quartile group than in the other groups. In contrast, the proportion of those using non-steroidal anti-inflammatory drugs, ever smokers and mean alcohol consumption was higher in the lowest quartile group. Mean body mass index, total energy intake and age at menarche did not differ among the highest to lowest quartiles of isoflavone consumption.

Table 2 shows the age, sex and screening-period-adjusted and multivariable-adjusted ORs and 95% CIs of colorectal adenoma for quartile categories of dietary isoflavone intake. Dietary isoflavone intake was associated with a reduced risk of colorectal adenoma in men and women combined. This inverse association was not linear; however, rather, a similar decrease was seen across all quartiles other than the first, with multivariable-adjusted ORs and 95% CIs in the second, third and highest compared with the lowest quartile of 0.77 (0.57–1.04), 0.76 (0.56–1.02) and 0.70 (0.51–0.96), respectively (*P* for trend=0.03). Stratified analysis by sex showed a significant inverse association between isoflavone consumption and colorectal adenoma in women, and a non-significant inverse association in men. The multivariable-adjusted ORs (95% CIs) for women in increasing quartile intake categories were 0.53 (0.28–0.98), 0.44 (0.24–0.80) and 0.49 (0.27–0.90), respectively (*P* for trend=0.03).

We repeated the above analyses after excluding those who had changed their dietary habits in the past 5 years (*n*=393 subjects), primarily on the basis that the change might have been related to the presence of adenoma. Results were essentially the same as those above (data not shown). We also investigated the association between colorectal adenoma for quartile categories of dietary isoflavone intake after further adjustment of fibre, folate, calcium and vitamin D in the multivariate model and similar decreased risk of association was observed (data not shown). We further categorised the subjects into octiles. As in the quartile analysis, however, the inverse association was not linear in the octile groups; rather, the third to highest octiles showed a decrease in risk, with the pattern more apparent in women, in whom multivariable-adjusted ORs (95% CIs) in increasing octile categories were 0.67 (0.26–1.70), 0.41 (0.16–1.06), 0.44 (0.18–1.05), 0.40 (0.17–0.97), 0.30 (0.13–0.72), 0.40 (0.17–0.93) and 0.42 (0.18–0.97), respectively (*P* for trend=0.03).

Stratified analysis according to site, size and number of colorectal adenomas showed no difference in the association with isoflavone intake by adenoma characteristics in overall men and women (Table 3). We further tested for effect modification between isoflavone intake and other covariates (age, sex, cigarette smoking, alcohol drinking and body mass index) in colorectal adenoma through the addition of multiplicative interaction terms into the model, but found no statistically significant *P*-value for interaction (data not shown).

## Discussion

In this colonoscopy-based case–control study, we found a significant inverse association between dietary isoflavone intake and colorectal adenoma risk in a Japanese population. The inverse association was not linear; with a ceiling effect observed with higher intake, which implied conversely that a lower intake of dietary isoflavone was related to an increased risk.

To our knowledge, this is the first study of this subject. Because adenomas are much more common than cancers, we may have greater power to detect the isoflavone–adenoma association. To date, three studies have examined the association between dietary isoflavone intake and the risk of colon (Oba *et al*, 2007) or colorectal cancer (Cotterchio *et al*, 2006; Akhter *et al*, 2008), two in Japanese (Oba *et al*, 2007; Akhter *et al*, 2008) and one in a Caucasian population (Cotterchio *et al*, 2006). Although the former two showed no clear association, the latter demonstrated a linear inverse association between dietary isoflavone intake and colorectal cancer risk. Of importance, the Caucasian population consumed a relatively low level of isoflavones, with a cut-off point for the highest tertile of 1.097 *vs* 17.22 mg per day for the lowest octile in our study population; it therefore investigated the effect of lower intake levels, which we could not observe in detail in our study, and supported our finding that a lower intake of dietary isoflavone was related to an increase in risk.

Isoflavones may help prevent the development of colorectal neoplasm through their oestrogenic effect. In fact, several *in vitro* and *in vivo* experimental studies have suggested that phyto-oestrogens protect against colorectal cancer (Spector *et al*, 2003). Phyto-oestrogens distinctly interact with oestrogen receptors (ERs) in the normal human colon. Of the ER-*α* and ER-*β* oestrogen receptors, phyto-oestrogens bind with high affinity to ER-*β*, and consistently mediate signals to inhibit abnormal cellular proliferation in colon tumour cell lines by blocking tyrosine protein kinases, aromatase and DNA topoisomerases. Phyto-oestrogens also possess antioxidant activity, and inhibit cell-cycle progression and angiogenesis in endothelial cells (Lechner *et al*, 2005).

We found a stronger inverse association in women than in men, although effect modification by sex was not statistically significant. Nonetheless, this suggested difference by sex is of particular interest, because isoflavones may have a more important function in the prevention of colorectal adenoma in women than in men. Of the five of seven epidemiological studies of the association between soya product intake and colorectal neoplasm, which provided results for men and women separately (Marchand *et al*, 1997; Ho *et al*, 2006; Wakai *et al*, 2006; Oba *et al*, 2007; Akhter *et al*, 2008), three reported that the inverse association was more prominent among women (Marchand *et al*, 1997; Ho *et al*, 2006; Oba *et al*, 2007), whereas none reported that it was stronger among men. This sex difference in the effect of plant-derived oestrogen (isoflavones) on colorectal neoplasm warrants further investigation.

Our study has several methodological advantages over previous studies. First, we used a validated FFQ and calculated isoflavone intake from a variety of dietary soya sources. Many different foods contribute to isoflavone intake, and no single soya food category predominates. Moreover, our subjects had relatively large isoflavone intake with substantial variation, making them suitable for the investigation of this association. Second, subjects answered the questionnaire before diagnosis, likely minimising the possibility of recall bias.

Several limitations also warrant mention. First, although all examinees underwent total colonoscopy, colorectal adenoma cases were diagnosed according to pit-pattern classification and were not confirmed histologically. Given an overall accuracy of pit-pattern diagnosis of approximately 90% *vs* data from our institute showing 95% accuracy, our analysis may have included a few false-positive cases (Sano *et al*, 2005). Magnifying chromoendoscopy is nevertheless a feasible and efficient method of determining neoplastic lesions such as adenoma and the validity of its inter- and intraobserver consistency has been confirmed (Haenszel *et al*, 1973; Fu *et al*, 2004). Second, the presence of adenoma might have been associated with changes in dietary habits; however, the sensitivity analysis revealed that such reverse association was less likely. Finally, isoflavone intake and other variables were based on self-administered questionnaires; some misclassifications of subjects were inevitable.

In summary, we found an inverse association between dietary isoflavone intake and the risk of colorectal adenoma in a Japanese population, which was not linear, but rather showed a ceiling effect associated with higher isoflavone intake; it was more prominent in women than in men, possibly reflecting an oestrogenic effect.

## Figures and Tables

**Table 1:** Demographic characteristics and lifestyle factors in men and women controls (*n*=697)

	**Quartiles of energy-adjusted intake of total isoflavones (mg per day)**				
	**Lowest**	**Second**	**Third**	**Highest**	
	**(<24.77)**	**(24.77 to <39.73)**	**(39.73 to <62.41)**	**(⩾62.41)**	
	**(*n*=174)**	**(*n*=175)**	**(*n*=174)**	**(*n*=174)**	***P-*value^a^**
*Sex*					
Men, *n* (%)	141 (81.0)	120 (68.6)	105 (60.3)	94 (54.0)	<0.01
Women, *n* (%)	33 (19.0)	55 (31.4)	69 (39.7)	80 (46.0)	
Age, mean (years)	59.2 (5.6)	59.1 (6.5)	60.7 (5.6)	60.4 (5.9)	0.02
Supplement use, yes (%)	71 (40.8)	72 (41.1)	89 (51.2)	91 (52.3)	0.04
Family history of colorectal cancer, n (%)	21 (12.1)	21 (12.0)	29 (16.7)	15 (8.6)	0.15
Non-steroidal anti-inflammatory drug use, n (%)	19 (10.9)	8 (4.6)	8 (4.6)	17 (9.8)	0.04
					
*Ever smoker, n (%)*	105 (60.3)	92 (52.6)	74 (42.5)	67 (38.5)	<0.01
Mean cigarettes (pack-years)	31.0 (27.8)	26.4 (24.5)	27.2 (21.6)	22.4 (21.0)	0.16
					
*Ever drinker, n (%)*	138 (79.3)	133 (76.0)	127 (73.0)	128 (73.6)	0.51
Mean alcohol (g per week ethanol)	218.4 (226.2)	201.0 (197.3)	158.1 (177.9)	140.0 (156.8)	<0.01
Body mass index, mean (kg m^*−2*^*)*	23.1 (2.6)	22.8 (3.0)	23.1 (2.7)	23.1 (3.0)	0.79
Physical activity, mean (MET h per day)	35.7 (6.8)	35.6 (7.0)	36.7 (7.1)	37.6 (9.2)	0.06
Total energy intake, mean (kcal per day)	1774.4	1900.0	1906.2	1839.1	0.11
Total isoflavone intake, mean (mg per day)^b^	17.3	31.4	49.4	78.5	<0.01
Age at menarche, mean (years)	13.4 (1.7)	13.1 (1.2)	13.2 (1.5)	13.3 (1.4)	0.72
Postmenopausal status, n (%)	31 (93.9)	47 (85.5)	64 (92.8)	70 (87.5)	0.43
Postmenopausal hormone use, n (%)	3 (9.1)	10 (18.2)	8 (11.6)	8 (10.0)	0.47

Mean values with standard deviations (s.d.'s) in parentheses for continuous variables; number of subjects with percentages in parentheses for categorical variables.

a *P*-values from analysis of variance (ANOVA) test for continuous variables; *χ*^2^-test for categorical variables.

b Energy adjusted.

**Table 2:** Logistic regression models of the effect of energy-adjusted intake of total isoflavones on colorectal adenoma

	**Quartiles of energy-adjusted intake of total isoflavones (mg per day)**				
	**Lowest**	**Second**	**Third**	**Highest**	
	**(<24.77)**	**(24.77 to <39.73)**	**(39.73 to <62.41)**	**(⩾62.41)**	***P*-value for trend**
*Men and women*					
Controls	174	175	174	174	
Cases	226	172	168	155	
OR (95% CI)^a^	1.00 (Reference)	0.75 (0.56–1.00)	0.73 (0.54–0.98)	0.67 (0.50–0.90)	<0.01
OR (95% CI)^b^	1.00 (Reference)	0.77 (0.57–1.04)	0.76 (0.56–1.02)	0.70 (0.51–0.96)	0.03
					
*Men*					
Controls	141	120	105	94	
Cases	173	119	114	86	
OR (95% CI)^c^	1.00 (Reference)	0.78 (0.55–1.10)	0.84 (0.59–1.20)	0.70 (0.49–1.02)	0.09
OR (95% CI)^d^	1.00 (Reference)	0.80 (0.56–1.14)	0.89 (0.62–1.28)	0.74 (0.50–1.09)	0.18
					
*Women*					
Controls	33	55	69	80	
Cases	53	53	54	69	
OR (95% CI)^e^	1.00 (Reference)	0.59 (0.33–1.05)	0.44 (0.25–0.75)	0.56 (0.33–0.96)	0.03
OR (95% CI)^f^	1.00 (Reference)	0.53 (0.28–0.98)	0.44 (0.24–0.80)	0.49 (0.27–0.90)	0.03

a Adjusted for age (40–49, 50–54, 55–59, 60–64, ⩾65 years), sex and screening period (first or second).

b Adjusted for age (40–49, 50–54, 55–59, 60–64, ⩾65 years), sex, screening period (first or second), family history of colorectal cancer (yes or no), cigarette smoking (never, ever ⩽20, ever 21–40, ever >40 pack-years), alcohol consumption (never, ever <150, ever 150–299, ever ⩾300 g per week ethanol), body mass index (<21, 21–22, 23–24, ⩾25 kg m^−2^), physical activity (<31.50, 31.50 to <34.55, ⩾34.55 MET h per day based on controls), supplement use (yes or no) and non-steroidal anti-inflammatory drug use (yes or no).

c Adjusted for age (50–54, 55–59, 60–64, ⩾65 years) and screening period (first or second).

d Adjusted for age (50–54, 55–59, 60–64, ⩾65 years), screening period (first or second), family history of colorectal cancer (yes or no), cigarette smoking (never, ever ⩽20, ever 21–40, ever >40 pack-years), alcohol consumption (never, ever <150, ever 150–299, ever ⩾300 g per week ethanol), body mass index (<21, 21–22, 23–24, ⩾25 kg m^−2^), physical activity (<31.50, 31.50 to <34.55, ⩾34.55 MET h per day based on controls), supplement use (yes or no) and non-steroidal anti-inflammatory drug use (yes or no).

e Adjusted for age (40–49, 50–54, 55–59, 60–64, ⩾65 years) and screening period (first or second).

f Adjusted for age (40–49, 50–54, 55–59, 60–64, ⩾65 years), screening period (first or second), family history of colorectal cancer (yes or no), cigarette smoking (never, ever ⩽20, ever 21–40, ever >40 pack-years), alcohol consumption (never, ever <150, ever 150–299, ever ⩾300 g per week ethanol), body mass index (<21, 21–22, 23–24, ⩾25 kg m^−2^), physical activity (<31.50, 31.50 to <34.55, ⩾34.55 MET h per day based on controls), supplement use (yes or no), non-steroidal anti-inflammatory drug use (yes or no), age at menarche (<12, 12–13, ⩾14 years), menopausal status (pre or post) and current use of female hormones (user or non-user).

**Table 3:** Logistic regression models of the effect of energy-adjusted intake of total isoflavones on site, size and number of colorectal adenoma in men and women

	**Quartiles of energy-adjusted intake of total isoflavones (mg per day)**				
	**Lowest**	**Second**	**Third**	**Highest**	
	**(<24.77)**	**(24.77 to <39.73)**	**(39.73 to <62.41)**	**(⩾62.41)**	***P*-value for trend**
Controls	174	175	174	174	
					
*Site of adenoma*					
Proximal colon	114	90	94	83	
OR (95% CI)^a^	1.00 (Reference)	0.75 (0.52–1.08)	0.77 (0.54–1.11)	0.69 (0.47–1.00)	0.07
Distal colon	82	67	59	52	
OR (95% CI)^a^	1.00 (Reference)	0.87 (0.58–1.30)	0.77 (0.51–1.17)	0.71 (0.46–1.09)	0.10
Rectum	30	15	15	20	
OR (95% CI)^a^	1.00 (Reference)	0.47 (0.24–0.93)	0.47 (0.24–0.93)	0.68 (0.36–1.31)	0.18
					
*Size of adenoma*					
<5 mm	125	93	97	87	
OR (95% CI)^a^	1.00 (Reference)	0.77 (0.54–1.09)	0.79 (0.55–1.12)	0.70 (0.48–1.00)	0.07
⩾5 mm	101	79	70	69	
OR (95% CI)^a^	1.00 (Reference)	0.74 (0.51–1.08)	0.67 (0.45–0.98)	0.73 (0.49–1.08)	0.08
					
*Number of adenomas*					
1 Adenoma	132	85	101	94	
OR (95% CI)^a^	1.00 (Reference)	0.64 (0.45–0.91)	0.73 (0.52–1.03)	0.67 (0.47–0.95)	0.05
>1 Adenomas	94	87	67	61	
OR (95% CI)^a^	1.00 (Reference)	0.96 (0.65–1.41)	0.72 (0.48–1.08)	0.74 (0.49–1.12)	0.07

a Adjusted for age (40–49, 50–54, 55–59, 60–64, ⩾65 years), sex, screening period (first or second), family history of colorectal cancer (yes or no), cigarette smoking (never, ever ⩽20, ever 21–40, ever >40 pack-years), alcohol consumption (never, ever <150, ever 150–299, ever ⩾300 g per week ethanol), body mass index (<21, 21–22, 23–24, ⩾25 kg/m^2^), physical activity (<31.50, 31.50 to <34.55, ⩾34.55 MET h per day based on controls), supplement use (yes or no) and non-steroidal anti-inflammatory drug use (yes or no).

## References

[bib1] Akhter M, Inoue M, Kurahashi N, Iwasaki M, Sasazuki S, Tsugane S, Japan Public Health Center-Based Prospective Study Group (2008) Dietary soy and isoflavone intake and risk of colorectal cancer in the Japan public health center-based prospective study. Cancer Epidemiol Biomarkers Prev 17: 2128–2135 18708407 10.1158/1055-9965.EPI-08-0182

[bib2] Arai Y, Watanabe S, Kimira M, Shimoi K, Mochizuki R, Kinae N (2000) Dietary intakes of flavonols, flavones, and isoflavones by Japanese women and the inverse correlation between quercetin intake and plasma LDL cholesterol concentration. J Nutr 130: 2243–2250 10958819 10.1093/jn/130.9.2243

[bib3] Brown CC, Kipnis V, Freedman LS, Hartman AM, Schatzkin A, Wacholder S (1994) Energy adjustment methods for nutritional epidemiology: the effect of categorization. Am J Epidemiol 139: 323–338 8116608 10.1093/oxfordjournals.aje.a117000

[bib4] Chlebowski RT, Wactawski-Wende J, Ritenbaugh C, Hubbell FA, Ascensao J, Rodabough RJ, Rosenberg CA, Taylor VM, Harris R, Chen C, Adams-Campbell LL, White E, Women's Health Initiative Investigators (2004) Estrogen plus progestin and colorectal cancer in postmenopausal women. N Engl J Med 350: 991–1004 14999111 10.1056/NEJMoa032071

[bib5] Cotterchio M, Boucher BA, Manno M, Gallinger S, Okey A, Harper P (2006) Dietary phytoestrogen intake is associated with reduced colorectal cancer risk. J Nutr 136: 3046–3053 17116718 10.1093/jn/136.12.3046 PMC1850957

[bib6] Fu KI, Sano Y, Kato S, Fujii T, Nagashima F, Yoshino T, Okuno T, Yoshida S, Fujimori T (2004) Chromoendoscopy using indigo carmine dye spraying with magnifying observation is the most reliable method for differential diagnosis between non-neoplastic and neoplastic colorectal lesions: a prospective study. Endoscopy 36: 1089–1093 15578300 10.1055/s-2004-826039

[bib7] Grodstein F, Newcomb PA, Stampfer MJ (1999) Postmenopausal hormone therapy and the risk of colorectal cancer: a review and meta-analysis. Am J Med 106: 574–582 10335731 10.1016/s0002-9343(99)00063-7

[bib8] Haenszel W, Berg JW, Segi M, Kurihara M, Locke FB (1973) Large-bowel cancer in Hawaiian Japanese. J Natl Cancer Inst 51: 1765–1779 4797262 10.1093/jnci/51.6.1765

[bib9] Hébert-Croteau N (1998) A meta-analysis of hormone replacement therapy and colon cancer in women. Cancer Epidemiol Biomarkers Prev 7: 653–659 9718216

[bib10] Ho SY, Schooling M, Hui LL, McGhee SM, Mak KH, Lam TH (2006) Soy consumption and mortality in Hong Kong: proxy-reported case-control study of all older adult deaths in 1998. Prev Med 43: 20–26 16631248 10.1016/j.ypmed.2006.03.007

[bib11] Hoshiyama Y, Sekine T, Sasaba T (1993) A case-control study of colorectal cancer and its relation to diet, cigarettes, and alcohol consumption in Saitama Prefecture, Japan. Tohoku J Exp Med 171: 153–165 8128484 10.1620/tjem.171.153

[bib12] Kimira M, Arai Y, Shimoi K, Watanabe S (1998) Japanese intake of flavonoids and isoflavonoids from foods. J Epidemiol 8: 168–175 9782673 10.2188/jea.8.168

[bib13] Kipnis V, Freedman LS, Brown CC, Hartman A, Schatzkin A, Wacholder S (1993) Interpretation of energy adjustment models for nutritional epidemiology. Am J Epidemiol 137: 1376–1380 8333419 10.1093/oxfordjournals.aje.a116647

[bib14] Lechner D, Kallay E, Cross HS (2005) Phytoestrogens and colorectal cancer prevention. Vitam Horm 70: 169–198 15727805 10.1016/S0083-6729(05)70006-6

[bib15] Linsalata M, Russo F, Notarnicola M, Guerra V, Cavallini A, Clemente C, Messa C (2005) Effects of genistein on the polyamine metabolism and cell growth in DLD-1 human colon cancer cells. Nutr Cancer 52: 84–93 16091008 10.1207/s15327914nc5201_11

[bib16] MacLennan SC, MacLennan AH, Ryan P (1995) Colorectal cancer and oestrogen replacement therapy. A meta-analysis of epidemiological studies. Med J Aust 162: 491–493 7746209

[bib17] Marchand LL, Hankin JH, Wilkens LR, Kolonel LN, Englyst HN, Lyu LC (1997) Dietary fiber and colorectal cancer risk. Epidemiology 8: 658–665 9345666 10.1097/00001648-199710000-00008

[bib18] Oba S, Nagata C, Shimizu N, Shimizu H, Kametani M, Takeyama N, Ohnuma T, Matsushita S (2007) Soy product consumption and the risk of colon cancer: a prospective study in Takayama, Japan. Nutr Cancer 57: 151–157 17571948 10.1080/01635580701274475

[bib19] Otani T, Iwasaki M, Ikeda S, Kozu T, Saito H, Mutoh M, Wakabayashi K, Tsugane S (2006) Serum triglycerides and colorectal adenoma in a case-control study among cancer screening examinees (Japan). Cancer Causes Control 17: 1245–1252 17111255 10.1007/s10552-006-0065-z

[bib20] Parkin DM, Whelan SL, Ferlay J, Teppo L, Thomas DB (eds) (2002) Cancer Incidence in Five Continents, Vol. 8, Lyon, France: IARC (IARC Scientific Publications No 155)

[bib21] Sano Y, Saito Y, Fu K-I, Matsuda T, Uraoka T, Kobayashi N, Ito H, Machida H, Iwasaki J, Emura F, Hanafusa M, Yoshino T, Kato S, Fujii T (2005) Efficacy of magnifying chromoendoscopy for the differential diagnosis of colorectal lesions. Digest Endosc 17: 105–116

[bib22] Spector D, Anthony M, Alexander D, Arab L (2003) Soy consumption and colorectal cancer. Nutr Cancer 47: 1–12 14769532 10.1207/s15327914nc4701_1

[bib23] Tsubono Y, Takamori S, Kobayashi M, Takahashi T, Iwase Y, Iitoi Y, Akabane M, Yamaguchi M, Tsugane S (1996) A data-based approach for designing a semiquantitative food frequency questionnaire for a population-based prospective study in Japan. J Epidemiol 6: 45–53 8795957 10.2188/jea.6.45

[bib24] Tsugane S, Kobayashi M, Sasaki S (2003) Validity of the self-administered food frequency questionnaire used in the 5-year follow-up survey of the JPHC Study Cohort I: comparison with dietary records for main nutrients. J Epidemiol 13: S51–S56 12701631 10.2188/jea.13.1sup_51 PMC9767699

[bib25] Wakai K, Hirose K, Matsuo K, Ito H, Kuriki K, Suzuki T, Kato T, Hirai T, Kanemitsu Y, Tajima K (2006) Dietary risk factors for colon and rectal cancers: a comparative case-control study. J Epidemiol 16: 125–135 16710081 10.2188/jea.16.125 PMC7603905

[bib26] Willett WC, Howe GR, Kushi LH (1997) Adjustment for total energy intake in epidemiologic studies. Am J Clin Nutr 65(Suppl): S1220–S1228 10.1093/ajcn/65.4.1220S 9094926

[bib27] Witte JS, Longnecker MP, Bird CL, Lee ER, Frankl HD, Haile RW (1996) Relation of vegetable, fruit, and grain consumption to colorectal adenomatous polyps. Am J Epidemiol 144: 1015–1025 8942431 10.1093/oxfordjournals.aje.a008872

[bib28] Yamaji T, Iwasaki M, Sasazuki S, Sakamoto H, Yoshida T, Tsugane S (2009) Methionine synthase A2756G polymorphism interacts with alcohol and folate intake to influence the risk of colorectal adenoma. Cancer Epidemiol Biomarkers Prev 18: 267–274 19124508 10.1158/1055-9965.EPI-08-0702

[bib29] Yamamoto S, Sobue T, Sasaki S, Kobayashi M, Arai Y, Uehara M, Adlercreutz H, Watanabe S, Takahashi T, Iitoi Y, Iwase Y, Akabane M, Tsugane S (2001) Validity and reproducibility of a self-administered food-frequency questionnaire to assess isoflavone intake in a Japanese population in comparison with dietary records and blood and urine isoflavones. J Nutr 131: 2741–2747 11584098 10.1093/jn/131.10.2741

